# Thermal Excitation of Gadolinium-Based Contrast Agents Using Spin Resonance

**DOI:** 10.1371/journal.pone.0158194

**Published:** 2016-06-24

**Authors:** Steven C. Dinger, Peter Fridjhon, David M. Rubin

**Affiliations:** 1 School of Electrical & Information Engineering, University of Witwatersrand, Johannesburg, Gauteng, South Africa; 2 School of Statistics & Actuarial Science, University of Witwatersrand, Johannesburg, Gauteng, South Africa; Martin-Luther-Universität Halle-Wittenberg, GERMANY

## Abstract

Theoretical and experimental investigations into the thermal excitation of liquid paramagnetic contrast agents using the spin resonance relaxation mechanism are presented. The electronic spin-lattice relaxation time *τ*_1e_ of gadolinium-based contrast agents, which is estimated at 0.1 ns, is ten orders of magnitude faster than the relaxation time of protons in water. The shorter relaxation time is found to significantly increase the rate of thermal energy deposition. To the authors’ knowledge this is the first study of gadolinium based contrast agents in a liquid state used as thermal agents. Analysis shows that when *τ*_1e_ and other experimental parameters are optimally selected, a maximum theoretical heating rate of 29.4 °C.s^−1^ could be achieved which would suffice for clinical thermal ablation of neoplasms. The experimental results show a statistically significant thermal response for two out of the four contrast agents tested. The results are compared to the simulated estimates via analysis of a detailed model of the system. While these experimentally determined temperature rises are small and thus of no clinical utility, their presence supports the theoretical analysis and strongly suggests that the chemical structure of the selected compounds plays an important role in this mechanism of heat deposition. There exists an opportunity for the development of alternative gadolinium-based compounds with an order of magnitude longer *τ*_1e_ in a diluted form to be used as an efficient hyperthermia agent for clinical use.

## Introduction

Currently no literature or evidence exists on using paramagnetic gadolinium-based contrast agents as hyperthermia agents. A large body of literature and research exists on using a variety of different materials and modalities for thermal treatment and as hyperthermia agents [1–3]. Brownian and Néel relaxation are some of the main mechanisms for heat deposition when using magnetic fluids or nano-particles [2]. The literature shows that super-paramagnetic iron oxide nano-particles are promising hyperthermia agents in both pharmaceutical and industrial sectors [2]. The thermal ablation of tumour cells using other clinical modalities such as high-intensity focused ultrasound (HIFU) is an example of a non-invasive hyperthermia treatment [4]. The HIFU technique however has associated technical problems which limits its effective use in many clinical situations [5]. An investigation into magnetic resonance imaging (MRI) localized tissue heating using the spin relaxation of protons in water was performed in 1984 by Parker [6]. The Parker 1984 paper is the first investigation into whether or not magnetic resonance of spin particles could be used as a heating modality. Parker showed using the power density at large rf magnetic field strengths ($\gamma^{2}B_{1}^{2}\tau_{1}\tau_{2}\gg 1$), given by Eqs 1 and 2, that it would take approximately two years to raise a 1 ml sample of water by one degree Celsius.

$$P_{\text{rf}}=\frac{N\gamma^{2}\hbar^{2}B_{0}^{2}}{4kT\tau_{1}},$$ (1)

$$P_{\text{rf}}≅3\times 10^{-9}\;\frac{B_{0}^{2}}{\tau_{1}}\;\;\text{J}.{\text{s}}^{-1}.\text{m}{\text{l}}^{-1},$$ (2)

where the proton density *N* = 0.66 × 10^23^ protons.ml^−1^, proton gyromagnetic ratio *γ* = 2.6752 × 10^8^ s^−1^.T^−1^, reduced Planck’s constant ℏ = 1.0546 × 10^−34^ J.s, Boltzmann constant *k* = 1.38 × 10^−23 ^J.K^−1^, body temperature *T* = 310 K, *B*_0_ = 1 T is the DC magnetic field density, *τ*_1_ = 0.1 s the proton relaxation time and *P*_rf_ the energy density transfer rate from the spin system into the liquid sample. The low heating rate obtained using protons in the water molecule is not practical for clinical use and hence there is clearly a necessity for further investigation into suitable spin particles.

The heating rate using the magnetic resonance of protons in water molecules is a problem mainly due to the associated spin properties of protons. The proton spins are relatively shielded in the water molecule and as a result exhibit long relaxation times. The spin relaxation time of protons in water is ideal for imaging purposes as they exhibit long free induction decays and can be excited using reasonable pulse durations. As seen in Eq (2) the spin-lattice relaxation time *τ*_1_ is an important parameter as it determines the rate at which thermal energy is deposited into the lattice.

The electron has a gyromagnetic ratio *γ* which is three orders of magnitude larger than that of a proton. The difference in magnitude is a result of the mass of the particles [7]. The electron being much less massive than a proton gives the electron a much larger gyromagnetic ratio, and hence a larger magnetic dipole moment. At an arbitrary external magnetic field strength *B*_0_, there are a greater number of electrons in a lower energy state compared with that of protons [8], as shown for the steady-state population ratio given by

$$\begin{array}{cc} \frac{N_{1}}{N_{2}} & =e^{\frac{\hbar \gamma B_{0}}{kT_{0}}}. \end{array}$$ (3)

A theoretical investigation is therefore presented into whether or not electrons could be used, as opposed to protons, to increase the heating rate.

## Theory

### Gadolinium

Gadolinium is part of the lanthanide or ‘rare-earth’ ions and has the highest number of unpaired electrons in its 4*f* orbital compared to any other element. As a result of this unique arrangement of electrons, the magnetic moment of the Gd^3+^ is relatively high (∼7.9 *μ*_*B*_) compared to other non-lanthanide elements [9]. The aquated Gd^3+^ ion is toxic in the blood, and for medical use is usually sequestered by chelation with multidentate ligands. The gadolinium chelates which are in current medical use differ in structural form; the main features are linear or macrocyclic and ionic or non-ionic [10].

### Spin-lattice relaxation

The mechanism by which energy is exchanged between the paramagnetic ions, known as the spin-system, and the electromagnetic thermal reservoir together with the surroundings (lattice vibrations) is explained. The relaxation of the spin-system occurs primarily through electron-spin flips induced by dynamic interactions with the surrounding environment, known as the ‘lattice’ [11]. The approach of *τ*_1e_ to zero implies an instantaneous energy exchange between the electromagnetic reservoir and the lattice via the spin-system.

It is the modulation of the zero-field splitting (ZFS) parameters which is responsible for the relaxation of the electrons in gadolinium complexes. The spin-lattice relaxation time of Gd^3+^ complexes is characterised by a quasi mono-exponential decay, as described by Rast et al [12], with a unique relaxation time *τ*_1e_ [12]. Using this static and transient ZFS model, Rast et al. were able to reproduce the Atsarkin measurement results for *τ*_1*e*_ [12, 13].

Referring to the literature it appears that the interchange of co-ordination geometry of DOTA and its stereoisomers does not play a significant role in modulating the ZFS parameters [14]. The parameters are most likely modulated by solvent collisions, rotational diffusion and vibrations of the complex [14]. The ZFS parameters seem to be correlated to the solution structure of the complexes which differ depending on the ligand used, such as macrocyclic ligands (DOTA) versus acyclic ligands (DTPA). An empirical rule therefore exists which correlates the structure of the complexes in solution to the ZFS parameters, and hence the observed differences in electronic relaxation times [15].

The relaxation time therefore depends on the type of chelation of gadolinium, as the molecular structure can result in a different symmetry and stereochemical rigidity. An example of this difference exists between Dotarem (Gd-DOTA) and Magnevist (Gd-DTPA) [15]. The DOTA chelate forms an axially symmetrical, macrocyclic and rigid structure around the Gd^3+^ ion, resulting in a six times longer *τ*_1e_ for the Gd-DOTA complex compared to the asymmetrical, linear and flexible Gd-DTPA molecule [15]. At low magnetic fields (*B*_0_ = 0.01 T) the electron spin relaxation time of the Gd-DOTA complex is estimated to be *τ*_1e_ = 0.1 − 0.2 ns for temperatures near *T* = 298 K [12].

### Spin-power

The energy released by the spin-system into the lattice per unit time is termed ‘spin-power’. In the presence of a DC magnetic field the spin-system absorbs energy from the rf (radio-frequency) field, which is created by a resonator, through a phenomenon called spin resonance. The energy is then lost to the lattice through a process called spin-lattice relaxation, which is characterised by a relaxation time *τ*_1e_. The rf power density absorbed by a non-saturated spin-system as derived by Slichter [7] and Weil [11], is given by

$$\begin{array}{cc} P_{\text{rf}} & =n_{0}\hbar \omega_{0}\frac{w_{e}}{1+2w_{e}\tau_{1e}}, \end{array}$$ (4)

where *w*_*e*_ is the induced electronic transition probability and is defined as function of frequency by

$$\begin{array}{cc} w_{e}\left(\omega_{0}\right) & =\frac{\pi }{2}\gamma^{2}B_{1}^{2}\left(S+m_{S}\right)\left(S-m_{S}+1\right)g\left(\omega_{0}\right). \end{array}$$ (5)

The secondary spin quantum number *m*_*S*_ ranges from −*S* to *S* in unit intervals with the Lorentzian *g*(*ω*_0_) representing the line-shape function normalised to unit area [7]. At the resonant angular frequency *ω*_0_ the lineshape function is

$$\begin{array}{cc} g(\omega_{0}) & =\frac{\tau_{2e}}{\pi }. \end{array}$$ (6)

The parameter *τ*_2e_ is the spin-spin relaxation time constant and represents the lifetime of the phase coherence among the spins. Given that the macroscopic electronic transition probability equation is applicable to substances where all the transitions occur at the same frequency [8], the states can be summed over all transitions using the following relation

$$\overset{S}{\underset{-(S-1)}{\sum }}(S+m_{S})(S-m_{S}+1)=\frac{2}{3}S(S+1)(2S+1),$$ (7)

$$w_{e}(\omega_{0})=\frac{1}{3}\gamma^{2}B_{1}^{2}S(S+1)(2S+1)\tau_{2\text{e}}.$$ (8)

The steady-state population difference *n*_0_ between two spin-states, *m*_*S*_ and *m*_*S*_ + 1, is given by

$$\begin{array}{cc} n_{0} & =\frac{2NS\left(S+1\right)\hbar \gamma B_{0}}{3kT}. \end{array}$$ (9)

Substituting Eqs (8 and 9) into Eq (4) results in the rf power absorbed by the spin-system per unit volume as

$$\begin{array}{cc} P_{\text{rf}} & =\frac{2N\gamma^{2}\hbar^{2}S\left(S+1\right)B_{0}^{2}w_{e}}{3kT(1+2w_{e}\tau_{1e})}. \end{array}$$ (10)

The parameters in Eq (10), for a particular case, are given in Table 1.

**Table 1:** Experimental values used to calculate the spin-power and resulting temperature rate, with the electronic spin relaxation times *τ*_1*e*_ and *τ*_2*e*_ obtained from Rast and Atsarkin [12, 13].

Parameter	Value	Units
*γ*	1.7608592 × 10^11^	rad.s^−1^.T^−1^
*N*	3.011 × 10^20^	number of Gd atoms per ml
ℏ	1.0545717 × 10^−34^	J.s.rad^−1^
*S*	7/2	resultant spin angular momentum
*B*_0_	30.6	mT
*k*	1.3806503 × 10^−23^	J.K^−1^
*T*	310.15	K
*τ*_1e_	0.1	ns
*τ*_2e_	0.1	ns
*B*_1_	1.5	mT
*w*_*e*_(*ω*_0_)	2.93 × 10^8^	s^−1^

The magnetic field *B*_1_ was estimated using simulations at the maximum 50 W rf amplifier power. The saturation term at this magnetic field strength is $\gamma^{2}B_{1}^{2}\tau_{1}{}_{\text{e}}\tau_{2}{}_{\text{e}}\ll 1$, and as a result the system is undersaturated [16]. The number of spins and relaxation times are estimated for the Dotarem solution, which has a concentration of 0.5 mmol.ml^−1^ and a density of *ρ*_*v*_ = 1.1753 g.ml^−1^. Solving Eq (10) using the parameter values in Table 1 and the unit ml for volume yields

$$\begin{array}{cc} P_{\text{rf}} & =65.99\;W.{\text{ml}}^{-1}. \end{array}$$ (11)

The unexpectedly large *P*_rf_ value suggests that the parameters require further adjustment in order to align with experimental results. The rf power absorbed by the spin-system is released into the lattice as heat, and thus the temperature rate in the substance, under adiabatic conditions, is solved using the following equations

$$\Delta Q_{h}=mC\Delta T,$$ (12)

$$\frac{dT}{dt}=\frac{P_{\text{rf}}}{\rho_{v}C}.$$ (13)

Assuming a heat capacity of *C* = 4.18 J.g^−1^.K^−1^ for the Dotarem solution, and substituting Eq (11) into Eq (13) results in a temperature rate of

$$\begin{array}{cc} \frac{dT}{dt} & =13.43\;{}^{\circ}C.s^{-1}. \end{array}$$ (14)

The electron spin-relaxation time *τ*_1*e*_ is a critical parameter in the spin-thermal system as it effectively determines the efficiency at which the absorbed rf energy is converted into thermal energy within the lattice. The implied assumption that *τ*_1*e*_ ≈ *τ*_2*e*_, which is only true for frequencies below 5 GHz, is validated from the direct measurements and predictions made by Atsarkin et al. [13]. The Atsarkin article also shows that for DOTA type aqueous complexes, *τ*_1*e*_ varies between 0.1 ns and 1 ns for 100 MHz − 10 GHz.

**Fig 1:**
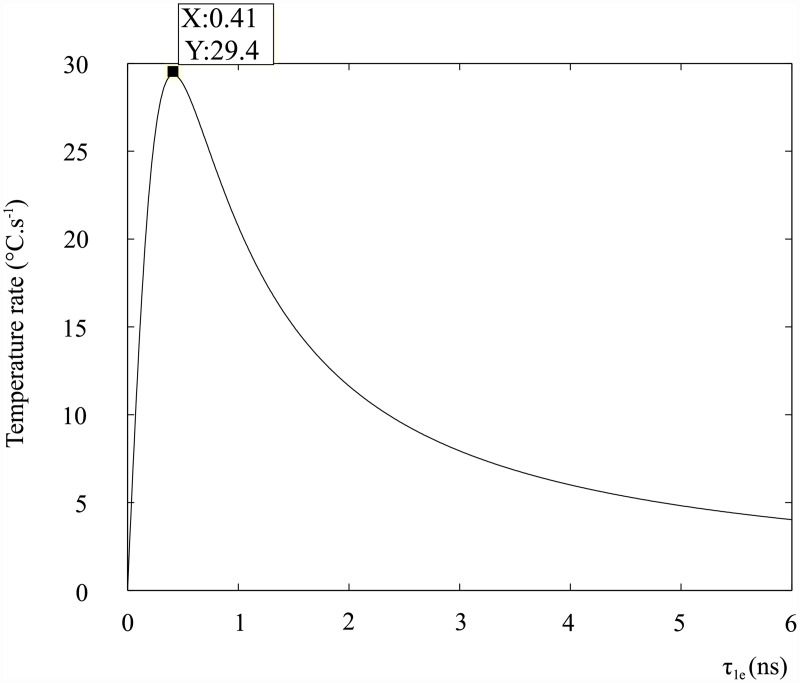
Temperature rate of the DOTA complex as a function of the spin-lattice relaxation time *τ*_1e_.

The maximum temperature rate as a function of the spin-lattice relaxation time *τ*_1e_, using the parameters in Table 1, is shown in Fig 1.

The plot shows that an optimal relaxation time exists, that is to say the spin-system can deliver heat efficiently to the lattice around some *τ*_1e_ for the given parameters. If *τ*_1e_ is too fast, the absorption lineshape is broad and the energy levels are widely distributed which results in a reduced spin-power. If *τ*_1e_ is too slow the spin energy is not released into the lattice in an adequate time and saturation is reached, which results in the spin power transfer being significantly reduced.

The theoretical maximum temperature rate obtained using spin resonance in contrast agents is comparable to current non-invasive tumour ablation treatments, such as HIFU, which can provide up to 14.3 °C.s^−1^ [17]. The optimal *τ*_1e_ relaxation time is approximately four times higher than the *τ*_1e_ of most contrast agents. Redesigning or modifying current contrast agents is therefore necessary in order to achieve larger temperature rates.

## Materials and Methods

**Fig 2:**
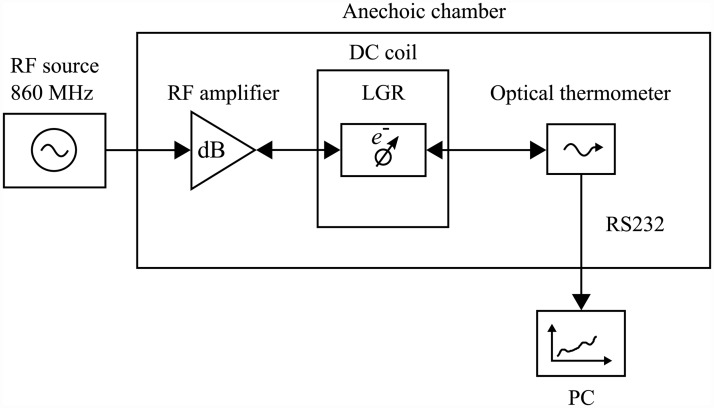
System block diagram of experimental setup.

The system block diagram of the experimental setup is illustrated in Fig 2.

The generator produces a 860 MHz continuous wave signal which is amplified by the rf amplifier to transmitted powers in the range of 1 − 4 W. The resulting magnetic field values *B*_1_, which were simulated and verified experimentally, ranged from 0.4 − 0.6 mT.

The resonant frequency of 860 MHz is selected in order to reduce the skin effect associated with high rf frequencies (> 1 GHz), and as a result lowered from the X-band frequencies. The lower frequency also helps to reduce the ohmic and induction losses in the sample. However, in order to maximise the spin resonance heating effect the frequency was increased above 100 MHz. Following iterative designs and simulations it was found that the optimal frequency range for experimentation was 800 − 900 MHz.

The amplified signal is fed into the loop-gap resonator (LGR) via a coupling loop. The LGR is a specialised resonator commonly used for electron paramagnetic resonance experiments [18]. The single-slot LGR is situated in a DC coil system which consists of eight concentric coils, with optimal turns ratios, that provide a homogeneous static magnetic field [19]. A design and construction technique for a four DC air-coil magnetic system, used for low-field EPR, is presented by Rinard et al [20].

The liquid sample is contained in a small PTFE tube (8 × 9 mm), which has an end-cap access port for the fibre optic thermometer probe. The fibre optic thermometer is capable of measuring temperatures, with a resolution and precision of 0.1°C, in large electric and magnetic field environments. The non-metallic fibre optic probe helps reduce the frequency-shift of the resonant cavity [21]. The experimental procedure for each substance is outlined as follows:

1. Switch on signal generator,
2. Raise sample temperature to steady-state value (≈ 37°C),
3. Monitor transmitted power of amplifier,
4. Switch DC coil on and record for 90 s,
5. Monitor transmitted power of amplifier,
6. Switch DC coil off and record for 90 s,
7. 30 s delay to achieve approximate steady-state,
8. Repeat measurements from step 3.

It was found during experimentation that an approximate steady-state could be achieved following the 90 s DC switch off interval. The additional 30 s delay allowed for the temperature deviation to be within the 0.1°C precision of the fibre optic thermometer.

The coil system, rf resonator and experimental materials were placed in an anechoic chamber to further reduce external noise sources. The ambient temperatures for all experiments were recorded, and ranged from 15 − 20°C. The building in which the anechoic chamber is situated, is air-conditioned with a relatively stable ambient temperature.

The ambient temperature deviation observed in the anechoic chamber was primarily due to heat produced by the DC coil system. Control experiments were therefore implemented in order to quantify the effect of heat produced by the DC coils.

There are two control substances and four test substances used in the experiments. The control substances (distilled water and saline) were chosen as they do not contain gadolinium and have a low and high electrical conductivity respectively. The test substances are the contrast agents, at 0.5 M concentrations, which are commonly used in clinical MRI scans. The substances are listed:

1. Distilled water,
2. Saline [0.9%],
3. MultiHance [Gd(BOPTA)^2−^],
4. Magnevist [Gd(DTPA)^2−^],
5. Dotarem [Gd(DOTA)^−^],
6. ProHance [Gd(HP-DO3A)],

with the chelating ligand, and resulting ionicity, of the contrast agents shown. As a result of the varying ionicity the electrical conductivity of each contrast agent differs, as shown in Table 2.

**Table 2:** Physical properties of tested substances and average experimental LGR values.

Substance	Cond. *σ*_*c*_ (S.m^−1^)*	$\epsilon_{r}^{\prime }$ **	Ave. frequency (MHz)	*s*_*f*_ (MHz)	Ave. rf power (W)	*s*_*p*_ (W)
Water	0.78 × 10^−3^	77.6	857.7	1.09	3.32	0.35
Saline	1.453	75.25	856.5	0.51	1.31	0.17
MultiHance	0.401	–	857.4	0.41	1.44	0.17
Magnevist	0.607	49.75	857.0	0.53	1.77	0.15
Dotarem	0.431	56.65	856.5	0.55	1.56	0.08
ProHance	0.0483	60.54	857.5	0.79	1.77	0.17

*Measured using a DC conductance meter (ECT estr11+) at room temperature.

**Real component of the complex permittivity, values obtained from Ogunlade.

It is observed from Table 2 that the conductivity does not only rely on the charge of the chelating agent but also on the excipients used in the compound. A thirty sample average of the frequency and input power to the loop-gap resonator, as well as the sample standard deviation (*s*_*f*_, *s*_*p*_) for each average value, is shown in Table 2.

The input power to the LGR for each substance was chosen such that the initial steady-state temperature was approximately 37°C, which matches human body core temperature and the normal operating temperatures for these clinical contrast agents. The rf input power required for a certain temperature is generally lower for higher electrically conductive solutions, however this is not the case for all contrast agents as the relative permittivity ($\epsilon_{r}^{\prime }$) affects the rf distribution inside the substance [22]. The higher permittivity is due to the lower solute concentration (g.l^−1^), for example ProHance (Gd[HP-DO3A]) which has the lowest contrast agent solute concentration of 279.4 g.l^−1^ [22].

## Results

**Fig 3:**
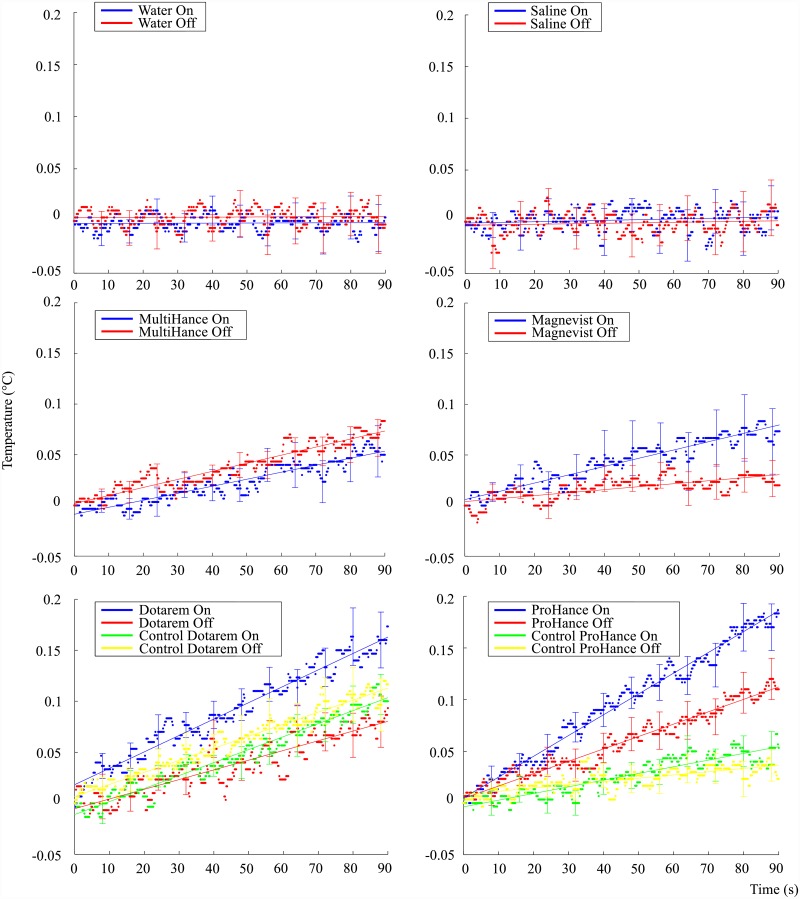
Mean temperature rise over a ninety second interval for the six tested substances, with each mean value obtained over thirty experiments and a linear regression line fitted. Standard error bars shown at every 8 s.

Multiple recordings at 0.2 s intervals were logged for each condition, with 30 experiments in total to accommodate the small temperature changes and measurement noise. The thirty sample average of the recorded temperature changes for each of the six substances, each with the DC magnetic field ‘On’ and ‘Off’ conditions, is shown in Fig 3. It is noted that each result is centered by subtracting the average initial value, approximately 37°C, from the dataset. The centralisation enables comparison between substances as well as the comparison of slopes for each substance.

The results show that no significant splitting or temperature difference occurs when the DC field is on or off for the control substances; water and saline. The test substances, or contrast agents, had varied responses with Dotarem and ProHance showing the most significant response to the DC field.

A control experiment was performed for Dotarem and ProHance whereby the bottom four coils of the DC magnetic coil system were connected in opposite direction to the upper four coils. The result of this reverse connection is that the net magnetic field over the sample space is approximately zero while the current, and therefore heat produced, in the coils remains the same. The heat loss from the coil remains the same in the control experiment due to the square of the current in the power equation.

The control experiment therefore tests whether the sample absorbs any heat released from the DC coil system and external environment. It is observed from Fig 3 that the control experiments for Dotarem and ProHance show no substantial splitting or difference in temperature rates when the DC field state is altered. The control experiment helps support the evidence that only the DC magnetic field is responsible for the on-off difference observed in the temperature responses of Dotarem and ProHance.

The mean value and standard error of the temperatures obtained from 30 repeated measurements are shown in Fig 3. It is noted from Fig 3 that the error bars for some of the contrast agents overlap at a few sample points. A statistical and quantitative analysis therefore of the amount of separation that exists between each substance is necessary. A linear regression analysis was performed and the models fitted to the data, as shown in Fig 3.

It is interesting to note that, except for the control substances, there exists a non-zero slope for the DC-Off condition. A possible reason for this residual effect is most likely due to the switch delay of approximately 6 − 12 *s*, which is mainly due to the inductance of the DC coil system and data acquisition. The spin system is therefore partially excited during the recorded switch-off interval. Future refinement of the experimental procedure is therefore necessary to eliminate this residual effect.

The contrast agent experiments, as well as the control experiments for Dotarem and ProHance, showed a temperature drift component during the Off condition. Apart from the switch delay, the non-zero average temperature drifts of the contrast agents and the control experiments are likely due to external factors such as the heat produced by the DC coil system and input power fluctuations of the resonator. The differences in conductivity, viscosity and specific heat capacity for each substance determines the magnitude of its temperature response to these external influences.

It is observed from Fig 3 that there is a small but noticeable sinusoidal pattern in the data. A possible cause of this pattern could be due to the conductivity changes in the sample due to heating/cooling resulting in a small resonance shift of the LGR. The input power oscillations of the resonator would then possibly promote the observed sinusoidal behaviour as the sample momentarily cools down and heats up again.

### Regression analysis

A first-order polynomial regression model is used to describe the data, given by the equation

$$\begin{array}{cc} y & =c+bx+\varepsilon , \end{array}$$ (15)

where *c* is the intercept, *b* the slope and *ε* the error term associated with the model [23]. The error term results from measurement device noise, human reproducibility, fluctuations in environmental conditions, substance variability, etc. Using the classical unweighted least-squares method the parameters of the straight line model are estimated.

The residual variance, also known as the squared standard error of the line of regression, is denoted $s_{y/x}^{2}$. Using two sets of data, with size *n*_1_ and *n*_2_, a comparison between their slopes, *b*_1_ and *b*_2_, is performed by using the regression models and their associated variance. Slope comparison of two regression lines results in the null hypothesis *H*_0_: *b*_1_ = *b*_2_ being tested, and is calculated using the Student’s t-test statistic [23]. The t-test statistic, which follows a *t*_*n*1+*n*2−4_ distribution, is given by

$$\begin{array}{cc} t & =\frac{b_{1}-b_{2}}{\sqrt{s_{(y/x),pool}^{2}\left(\frac{1}{\Sigma {\left(x_{i,1}-{\bar{x}}_{1}\right)}^{2}}+\frac{1}{\Sigma {\left(x_{i,2}-{\bar{x}}_{2}\right)}^{2}}\right)}}, \end{array}$$ (16)

with

$$\begin{array}{cc} s_{(y/x),pool}^{2} & =\frac{\left(n_{1}-2\right)s_{(y/x)1}^{2}+\left(n_{2}-2\right)s_{(y/x)2}^{2}}{n_{1}+n_{2}-4} \end{array}$$ (17)

The results of the linear regression fit and the comparison of slopes, between the 1 = ‘On/cOn’ and 2 = ‘Off/cOff’ states, are shown in Table 3.

**Table 3:** Slope values *b*, with subscript definitions 1 = On/cOn and 2 = Off/cOff states, and comparison results performed on experimental datasets.

Substance	Experiment	*b*_1_ (×10^−6^ °C.s^−1^)	*b*_2_ (×10^−6^ °C.s^−1^)	p-value
Water	On-Off	9	14	0.788
Saline	On-Off	66	48	0.475
MultiHance	On-Off	692	796	< 0.01
Magnevist	On-Off	820	295	< 0.01
Dotarem	On-Off	1607	951	< 0.01
	On-cOn	1607	1273	< 0.01
	cOn-cOff	1273	1179	< 0.01
ProHance	On-Off	2011	1198	< 0.01
	On-cOn	2011	646	< 0.01
	cOn-cOff	646	315	< 0.01

The On state represents current flow in the same direction for both DC coils, and the Off state represents no current flow in the DC coils. The cOn state represents current flow in opposite direction for top and bottom DC coils, and the cOff state represents no current flow in the DC coils. The samples size for each dataset is *n*_1_ = *n*_2_ = 451, which is due to the 0.2 s sampling time of the fibre optic thermometer.

The results show that the slopes are statistically significantly the same for the control substances; water and saline. The results also show that the slopes are statistically different for the contrast agents and the control experiments for Dotarem and ProHance. Although ProHance is shown to be significantly different between the cOn-cOff states, the actual cOn state slope value is three times smaller compared to the On state slope value.

The statistical significance obtained for the control on-off experiments is mainly due to the large number of samples, and therefore large degrees of freedom used in the comparison, as illustrated by the almost identical, yet significant, slope values of the Dotarem control results. Secondary contributors of significance are the aforementioned external influences of the heated DC coil system and resonator fluctuations. A qualitative interpretation of the slope values is that large separation exists between the on and off states of Magnevist, Dotarem and ProHance.

### Model analysis

It is noted from the regression analysis results that the net temperature rate for Dotarem and ProHance, using the difference in slope values of the linear regression model for the treatment DC-On and control DC-On, is 334.1 × 10^−6^ °C.s^−1^ and 1,364 × 10^−6^ °C.s^−1^ respectively. The experimental temperature rates are significantly lower than the theoretical prediction of 1.8 °C.s^−1^, which is obtained from Eq (13) when using similar parameters to experimentation i.e. *B*_1_ = 0.54 mT and *f* = 857 MHz. As a result of this discrepancy the spin-lattice relaxation time is in need of adjustment by further modelling in order to estimate its value correctly under these specific conditions. A thermal model of the sample, sample tube and fibre-optic probe system is given by

$${\dot{Q}}_{a}={\dot{Q}}_{l}+{\dot{Q}}_{s}-\frac{T_{w}-T_{a}}{R_{c}+R_{a}}-\frac{T_{w}-T_{t}}{R_{t}},$$ (18)

$${\dot{Q}}_{t}=\frac{T_{w}-T_{t}}{R_{t}}-\frac{T_{t}-T_{p}}{R_{p}},$$ (19)

$${\dot{Q}}_{p}=\frac{T_{t}-T_{p}}{R_{p}},$$ (20)

where ${\dot{Q}}_{a}$ is the net-thermal power in the contrast agent, ${\dot{Q}}_{l}$ is the liquid Ohmic-power loss, ${\dot{Q}}_{s}$ is the spin-power, *T*_*w*_ is the temperature of the substance, *T*_*a*_ is the ambient temperature, *R* is the thermal resistance (subscript *a*-1 mm air sleeve, *c*-PTFE sample container, *t*-PTFE outer fibre optic probe coating and *p*-polyimide inner fibre optic probe coating). The constant model parameters, other than the specific heat, were verified using a rf pulse response for water, since water has a well-known specific heat value. The rf pulse experiment involves switching on the rf power, without a DC magnetic field, for approximately 30 s (${\dot{Q}}_{l}>0\;W$) and then switching off the rf power for the remaining 60 s (${\dot{Q}}_{l}=0\;W$).

**Fig 4:**
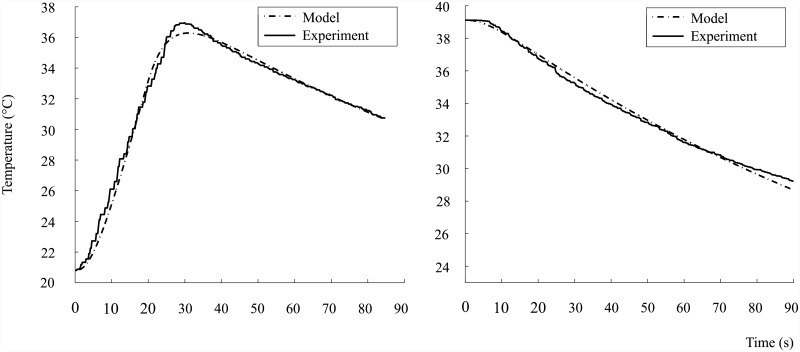
Experimental and model estimate for the specific heat capacity of ProHance using the average of the pulse (*C*_Δ_) and decay (*C*_*d*_) response.

The specific heat capacity of each contrast agent $\bar{C}$ is estimated using the average of the specific heat capacity for a rf pulse response *C*_Δ_ and the specific heat capacity for a negative-edge step response *C*_*d*_. An example of the two types of responses is shown for the ProHance solution in Fig 4.

The heat capacity of the substance is substituted into the sensible heat equation

$$\begin{array}{cc} T & =\frac{Q_{h}}{mC}, \end{array}$$ (21)

which determines the temperature of each substance or material in the set of model equations. The list of calculated and measured parameters used to determine the models in Fig 4, are shown in Table 4.

**Table 4:** Model parameters used to fit the pulse and decay responses of the ProHance solution.

Parameter	Description	Pulse Value	Decay Value	Unit(s)
${\dot{Q}}_{l}$	Ohmic power loss	0.646 (*t* < 30 s)	0	W
*T*_*a*_	Ambient temperature	290.8	288.0	K
*R*_*c*_	Thermal resistance of sample container	39.5	39.5	K.W^−1^
*R*_*a*_	Thermal resistance of air sleeve	163.7	163.7	K.W^−1^
*R*_*t*_	PTFE fibre probe outer coating	35.7	35.7	K.W^−1^
*R*_*p*_	Polyimide fibre probe inner coating	265.3	265.3	K.W^−1^
*m*_*t*_	Mass of PTFE probe outer coating	0.026	0.026	g
*m*_*p*_	Mass of polyimide probe inner coating	0.018	0.018	g
*m*_*w*_	Mass of ProHance sample	0.289	0.289	g
*C*_*t*_	Specific heat capacity of PTFE probe coating	1.01	1.01	J.g^−1^.K^−1^
*C*_*p*_	Specific heat capacity of polyimide probe coating	1.09	1.09	J.g^−1^.K^−1^

The minimum of the normalised root-mean-square error (NRMSE) is used to determine the best model estimate of the specific heat capacity value for each response, as shown in Table 5.

**Table 5:** Specific heat capacity of Dotarem and ProHance using pulse and decay modelled data.

Substance	*C*_Δ_ (J.g^−1^.K^−1^)	NRMSE (%)	*C*_*d*_ (J.g^−1^.K^−1^)	NRMSE (%)	$\bar{C}\;(J.g^{-1}.K^{-1})$
Dotarem	1.78	1.67	2.72	2.95	2.25
ProHance	2.1	2.52	2.36	2.57	2.23

**Fig 5:**
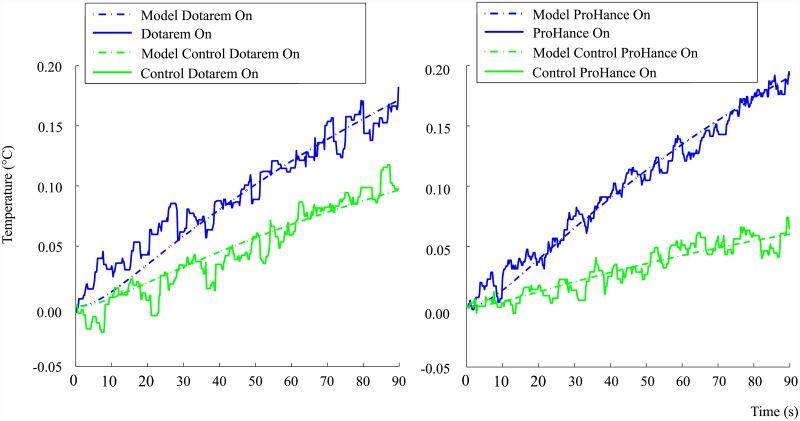
Model estimates for Dotarem and ProHance treatment-control condition responses.

Using the estimated parameters of the container and substances, the Ohmic-loss and spin-power are estimated for the treatment-on and control-on conditions for Dotarem and ProHance, as shown in Fig 5.

The heating rates were adjusted to minimise the normalised root-mean-square error, with the results shown in Table 6. Note that ${\dot{Q}}_{T}$ in Table 6 represents the total heat generated in the sample.

**Table 6:** Model estimate of total-power, Ohmic loss, spin-power and spin-lattice relaxation time *τ*_1e_ for Dotarem and ProHance.

Substance	${\dot{Q}}_{T}\;(W)$	NRMSE (%)	${\dot{Q}}_{l}\;(W)$	NRMSE (%)	${\dot{Q}}_{s}(W)$	*τ*_1e_ (*ps*)
Dotarem	0.1135	8.18	0.1081	7.36	0.005375	0.26
ProHance	0.1076	4.56	0.1032	9.97	0.004400	0.15

The spin-lattice relaxation time *τ*_1e_ is estimated from the modelled spin-power heating rates, experimental values for *B*_1_ and Eq (10), with the estimates shown in Table 6. It is noted from Table 6 that the estimated relaxation times are approximately three orders of magnitude smaller than the simulated estimate of 0.1 ns performed by Rast et al [12]. A reason for the discrepancy is most likely due to the high solution concentration effects enhancing the dipole-dipole interactions and as a result significantly decreasing the electronic relaxation times [24].

Another possible explanation for the observed differences is due to the short rotational correlation times, in the order of 0.1 ns, associated with the fast molecular tumbling rates of the Gd-based contrast agents [25]. The short rotational correlation time tends to broaden the spectral density function which enhances the relaxation processes, and as a result lowers the spin-relaxation time [26]. It is also generally accepted that the electronic spin-relaxation time decreases as the magnetic field strength and Larmor frequency decreases [25]. The exact magnetic field dependence of the electronic spin-relaxation time for Gd complexes in solution however is not well-known in the low-field region (*B*_0_ < 0.1 T) [25].

Under similar experimental conditions a molecule with a longer spin-lattice relaxation time would result in a larger spin-power and temperature change. In the current operating region the spin-power effect is approximately linear against relaxation time, as seen from Eq (10) for *τ*_1*e*,2*e*_ < 0.1 ns. The consequence of this linearity is that an order of magnitude increase in *τ*_1e_ will result in an order of magnitude increase in spin-power.

## Discussion

The research suggests that a measurable heating rate is achieved using the seven unpaired electrons present in gadolinium-based contrast agents, with Dotarem and ProHance having the most marked responses to the DC magnetic field. The other contrast agents MultiHance and Magnevist showed relatively small temperature responses, a result most likely due to the combined effect of a linear chemical ligand structure and high solution conductivity.

The origin of the discrepancy between *τ*_1e_ = 0.1 ns and the three orders lower value derived from our experiments remains unknown. There is a possible contribution of the dipole-dipole interaction between the Gd complexes at our high concentration that is not taken into account. The EPR spectrum of the Gd-complexes at low frequencies is also not well understood and is a possible cause of the observed low heating rate due to mismatching of transition energies. To exclude these omissions in the theory of the heating of the solution is therefore not possible.

Although the observed electron spin resonance heating rate is in the milli-Watt range, it is still significantly larger (167 000 times) compared to the heating rate obtained using protons as derived by Parker. The research therefore presents novel results which suggest that thermal energy can be deposited into a liquid paramagnetic system using electron spin resonance.

In order to achieve practical or clinical heating rates however, the spin-relaxation time should be increased to an optimal value by developing more suitable ‘contrast agents’. As can be appreciated from the research, many challenges exist in making spin resonance heating a viable and practical technology for non-invasive treatment of tumours, however it is hoped that the research presented here is the first step towards making this technology possible.
